# On the Wrong Track: Process and Content in Moral Psychology

**DOI:** 10.1111/mila.12001

**Published:** 2012-10-29

**Authors:** Guy Kahane

**Affiliations:** Faculty of Philosophy, Oxford University

## Abstract

According to Joshua Greene's influential dual process model of moral judgment, different modes of processing are associated with distinct moral outputs: automatic processing with deontological judgment, and controlled processing with utilitarian judgment. This article aims to clarify and assess Greene's model. I argue that the proposed tie between process and content is based on a misinterpretation of the evidence, and that the supposed evidence for controlled processing in utilitarian judgment is actually likely to reflect, not ‘utilitarian reasoning’, but a form of moral deliberation which, ironically, is actually in serious tension with a utilitarian outlook. This alternative account is further supported by the results of a neuroimaging study showing that intuitive and counterintuitive judgments have similar neural correlates whether or not their content is utilitarian or deontological.

The recent psychology of morality has a familiar narrative arc. Until the turn of the century, most philosophers and psychologists had assumed that moral judgment is based in reason. But then striking new evidence from psychology and neuroscience turned this traditional picture on its head. It turns out—so the story goes—that moral judgment is not based in reason or conscious reflection, but in immediate intuitions and emotion. Moreover, individuals rarely engage in deliberation when they make moral judgment, and even when they do, their reasoning merely aims to rationalize a pre-established intuitive conclusion. To shift to psychological terms, the emerging new consensus is that moral judgment is based almost exclusively in processes that are *automatic*—fast, effortless, and unconscious—and only rarely, if at all, in processes that are *controlled*—slower, effortful and involving explicit conscious thinking (Haidt, 2001, 2007, 2012; Greene and Haidt, 2002; for general discussion of such dual process models, see Stanovich, 1999; Evans, 2007).

This is an often-told tale, but it is hardly news to moral philosophers that intuition plays a central role in our moral lives. Moral philosophers *would* be surprised, however, and disturbed, to find out that our intuitions are not just starting points for ethical reflection, but also where it invariably *ends*. A pressing question, then, is whether moral judgment is ever also based in deliberation or ‘controlled processing’.

A growing body of research by Joshua Greene and his colleagues, and research influenced by it, has offered a striking answer to this question. While broadly confirming the view that automatic processing drives the moral judgments of most individuals, it also appears to show that there is a minority that not only engages controlled processing in moral decision-making, but also uses it to arrive at moral conclusions that, far from being mere rationalizations of prior intuitions, actually go *counter* to our intuitions (Greene *et al*., 2001; Greene *et al*., 2004; Greene, 2008; Paxton and Greene, 2010).

What is most striking about this research, however, is that it has tied these opposing modes of *processing* to moral judgments with opposing *contents*. For it appears to show that when the majority follows their immediate intuitions, the result is deontological in content, whereas when individuals do engage controlled reasoning, they arrive instead at contrary utilitarian conclusions.^1^ This dual process model of moral judgment makes intuitive sense. After all, it is notorious that utilitarianism has counterintuitive implications that many find repugnant.

Greene's dual process model is of considerable theoretical interest. But more is at stake here, since Greene and others have gone on to argue that this theory has dramatic ethical implications—that it offers support for utilitarianism, and thus for precisely these repugnant, counterintuitive implications (Greene, 2008; Singer, 2005.). It is not surprising that these normative claims, and the dual process that underpins them, have therefore attracted critical scrutiny (see e.g. Berker, 2009; Kahane and Shackel, 2008, 2010). But there is as yet no persuasive alternative view of the role of controlled processing in moral judgment that takes into account the full range of evidence.

In this article I will offer such an account. I will draw attention to several critical points of unclarity in Greene's dual process model. I argue that once these are probed, it emerges that the model is based on an implausible interpretation the evidence, and that the tie between psychological process and moral content assumed by Greene does not hold. I will argue instead that the role of controlled processing in moral psychology is to sustain deliberation that can sometimes lead to counterintuitive conclusions—which may or may not be utilitarian, depending on the context. In fact I will show that the evidence that is supposed to establish a tie between controlled processing and utilitarian reasoning is most likely to reflect a form of deliberation that is inherently *incompatible* with a utilitarian outlook. I will highlight key points where the empirical predictions of my account directly conflict with those made by Greene's model, and I will review evidence, including results from a recent neuroimaging study we have conducted (Kahane *et al.*, 2012), that seems to me to strongly favour my model.

## 1. Greene's Dual Process Model

### 1.1. Clarifying the Model

Greene's dual process model consists of two claims:

1. **DP1.** Deontological judgments are generated by automatic processing.
2. **DP2.** Utilitarian judgments are generated by controlled processing.

Notice first that these are distinct empirical claims. The first claim could be true even if all moral judgments were based in automatic processing, and vice versa. Importantly, we mustn't assume that evidence supporting one of these claims automatically supports the other.

The intended strength of these claims is not entirely clear. They plainly don't intend to assert some *necessary* connection between process and content. Needless to say, Greene's model won't be falsified if we find just one instance where a deontological judgment was produced by controlled processing. On the other hand, the model plainly doesn't merely claim that although controlled processing produces both deontological and utilitarian judgments, the latter output is just a little more frequent. The idea seems to be not only that utilitarian judgments are the *favoured outputs* of controlled processing, but also that contrary outputs are exceptions, even aberrations. Notice moreover that the model makes a claim about the *causal source* of each type of judgment. It is perfectly compatible with deontological judgments sometimes *engaging* controlled processing in an extensive way, so long as this processing plays a merely epiphenomenal role in the final output—so long as it is engaged merely to offer a rationalization of the initial intuition.^2^

Whatever their precise strength, the two claims that make up Greene's dual process model are clearly meant to be entirely general. They are meant to apply across moral contexts and domains. But most of the research taken to support the model has focused on so-called ‘trolley problems’ and similar scenarios where one person must be seriously harmed to save a greater number. For example, in the *Bystander* case, you can save five workers from being run over by a runaway trolley by diverting it to another track, where it will kill one worker. In the *Footbridge* case, you can again save five workers from being run over by a runaway trolley only by doing something that would lead to the death of another innocent person, except that here, to prevent the death of the five, you must push a large man onto the trolley's path, leading to his death. As much research shows, most people believe that it's right to divert the trolley in Bystander, but wrong to push the man in Footbridge (Cushman *et al*., 2006).

Such dilemmas, of course, relate to a rather unusual part of morality, and are merely one of numerous contexts in which utilitarianism clashes with common-sense intuitions.^3^ So we should be careful to distinguish—as many researchers do not—what we can call the Modest Dual Process Model, which endorses DP1 and DP2 *only* with respect to judgments about such trolley-style scenarios, and what we can call the Grand Dual Process Model, which makes these claims about deontological and utilitarian judgments in general. There is a considerable gap between the two models. It is important to keep in mind that although Greene clearly intends to defend the Grand Model (Greene, 2008), at present most of the evidence really at best supports the far weaker Modest Model.^4^ And notice that it is yet a *further* large step from the Grand Model to any kind of claim about the causal basis of explicit *philosophical theories* such as utilitarianism and Kantian ethics. Greene's normative arguments rely on this further step, which is at present highly speculative.

A further distinction: whether modest or not, the dual process model is a claim about the psychological *processes* underlying certain types of moral judgments. It is *not* a claim about the *factors* to which these judgments are sensitive.^5^ In earlier work, Greene suggested that deontological judgments in Bystander and Footbridge are responsive to the presence/absence of ‘personal’ harm. In more recent work, he presents evidence that it is responsive to the presence/absence of intention to harm coupled with ‘personal force’ (Greene *et al*., 2009). But these claims are independent of the dual process model; such a claim could be true and the dual process model false, and vice versa.

In what follows, I shall largely focus on the supposed link between utilitarian judgment and controlled processing (DP2). But let me begin with two brief remarks about the other half of the dual process model, the claim associating deontological judgment with automatic processing (DP1).

### 1.2. Not About Emotion and Cognition

It might seem surprising that in stating DP1, I did not present it as a claim about the *emotional* basis of deontological judgment. This omission is deliberate. Although questions about the role of automatic and controlled processing in moral judgments are often conflated with questions about the role of emotion and cognition, this is a mistake. To be sure, Greene also endorses:

(E) Deontological judgments are generated by *emotional* processing.
(C) Utilitarian judgments are generated by *cognitive* processing.

But although Greene doesn't always distinguish the above claims from DP1 and DP2, these are distinct sets of claims. This is not only because there is no agreed understanding of the rather vague common-sense distinction between emotion and cognition (Huebner *et al*., 2009). For even if we assume this intuitive distinction, it does not directly map onto the automatic/controlled processing distinction.

To start with, DP1 could be true even if (E) is false. After all, paradigmatic instances of automaticity—think here of linguistic intuitions—don't seem to involve emotion in any way. The current debate about emotion in moral judgment between e.g. Haidt and Greene on the one hand and Hauser and Mikhail on the other (cf. Huebner *et al*., 2008) is really about the involvement of emotion in automaticity. *Both* sides accept DP1; they are disagreeing only about (E) above. Notice that this point also means that (C) could be true but nevertheless refer to *automatic* processing. Moreover, although many researchers seem to assume that emotional processing implies automatic processing (so that (E) would at least imply DP1), even this is link is dubious. There are after all emotions, such as being torn between two opposing choices in a dilemmatic situation, that arise *only* in the context of effortful controlled processing. The common failure to distinguish between (DP1) and (E) often leads to confusion.

Not only isn't the emotion/cognition distinction distinct from the automatic/controlled distinction, it is not even clear that much hangs on it. It might have been important if it marked a distinction between primitive gut reactions and complex computation, as was sometimes implied by Greene's earlier work. But even on Greene's own current view, the purported automatic emotional responses that are supposed to underlie deontological judgment are rather complex, involving the attribution of rich intentional and causal properties (Greene *et al*., 2009). And there is considerable evidence that, emotional or not, these responses *are* extremely complex (Huebner, Hauser and Pettit, 2011)—indeed they appear to be *more* complex than a mere utilitarian cost-benefit analysis. Thus, and given that the empirical evidence for the exact role of emotion in deontological judgment is still contested and unclear (Huebner *et al*., 2008), I will largely set aside this aspect of Greene's view.

### 1.3. Deontology and Automatic Processing

Once we set aside the question of emotion, (DP1) isn't especially controversial, and I shall largely accept it in what follows. I'll accept it for the simple reason that it is not really a great surprise that the deontological distinction between the Bystander and Footbridge cases, and various other deontological distinctions and claims, are based in intuition.^6^ Again, it *would* be troubling if explicit deontological theories were no more than the rationalization of such intuitions, but there is little or no evidence at present for this further claim.^7^ But some appropriately weakened version of DP1 doesn't seem to me especially controversial, though we shall later see some ways in which DP1 needs to be seriously qualified.^8^

## 2. Are Utilitarian Judgments Uniquely Based in Controlled Cognitive Processing?

So let us turn to the second half of the dual process model, the claim tying utilitarian judgment with controlled processing. This is after all what is supposed to present utilitarianism is a more favourable light, and the point where Greene's model departs from the emerging consensus of pervasive moral automaticity.

### 2.1. The Evidence for the Link Between Utilitarian Judgment and Controlled Processing

We can begin by quickly surveying the evidence. Keep in mind that we need to distinguish here evidence for the Modest Model from evidence for the Grand Model, and evidence for DP1 and for DP2. Much of the evidence typically cited in support of the dual process model is really evidence for the modest version of DP1—actually, much of it is really just evidence that deontological judgments in trolley-like cases involve or are triggered by *emotion* (that is, evidence for (E) rather than for (DP1)). This includes neuroimaging evidence that responses to the Footbridge case and other ‘personal’ dilemmas are associated with greater activity in parts of the brain implicated in emotion compared to responses to the Bystander case and other ‘impersonal’ dilemmas (Greene *et al*., 2001; Greene *et al*., 2004), and evidence from lesion studies showing greater rates of utilitarian judgment in ‘personal’ dilemmas in patients with damage to the ventromedial prefrontal cortex (VMPFC) which is associated with loss of social emotion (Mendez *et al*., 2005; Koenigs *et al*., 2007; Ciaramelli *et al*., 2007; Moretto *et al*., 2009).

The available evidence for controlled processing in utilitarian judgment is more limited. Neuroimaging studies have reported that moral judgments in ‘impersonal’ dilemmas recruit greater activity in areas classically associated with cognitive processing (the right dorsolateral prefrontal cortex (DLPFC) and inferior parietal lobe) compared to ‘personal’ dilemmas (Greene *et al*., 2001; Greene *et al*., 2004), and evidence that utilitarian judgments in ‘difficult’ personal dilemma recruit more DLPFC and the dorsal anterior cingulate cortex (dACC) activation compared to contrary deontological judgments (Greene *et al*., 2004).^9^

The classic marker of the automatic/controlled contrast is differences in response times (RT)—controlled processing should take longer. Although Greene originally reported that utilitarian judgments in personal dilemmas were associated with greater RTs compared to deontological ones (Greene *et al*., 2001; Greene *et al*., 2004), these results have not held up when the stimuli used were better controlled (Moore *et al*., 2008; Greene *et al*., 2008; see also McGuire *et al*., 2009; Greene, 2009). However, another classical marker is interference through cognitive load: a competing task should affect only responses based in controlled processing. Greene *et al*. (2008) report that a cognitive load manipulation raised response times for utilitarian judgments but not for deontological judgments in ‘high conflict’ personal dilemmas. However, contrary to what Greene's model predicts, such a manipulation did not affect the rates of utilitarian judgment.

Finally, higher rates of utilitarian judgment were found to be associated with indirect measures of controlled processing, such as individual difference in a motivational tendency to prefer effortful cognition (Bartels, 2008), and greater working memory capacity (Moore *et al*., 2008). In addition, exposure of people to non-moral problems with counter-intuitive answers induced greater rates of utilitarian judgment (Paxton *et al*., 2012).^10^

Notice that the evidence I surveyed is only evidence for what I called the Modest Model:

1. **Modest DP2.** Utilitarian judgments in *trolley cases* are generated by controlled processing.

In fact, virtually all of it is evidence for an even *narrower* claim about the involvement of controlled processing in the *Footbridge* case and similar ‘personal’ dilemmas.

Although Greene does cite some further evidence for the involvement of emotion in deontological judgments in other domains, such as retributive punishment (Greene, 2008), I am not aware of any serious evidence that utilitarian judgments in other domains involves controlled processing.^11^ So at present it is a rather speculative inference from the existing evidence to the far grander claim that

1. **Grand DP2.** Utilitarian judgments are *generally* generated by controlled processing.

Having highlighted this obvious gap in the evidence for Greene's dual process model, in what follows I will argue that even the *modest* claim is highly problematic. It is problematic because it implicitly relies on a *further* inference that is far from obvious.

### 2.2. A Second Problematic Inference

Greene and others slide from the supposed empirical finding that:

(1) When individuals make utilitarian judgments in trolley cases, controlled processing plays a causal role in the generation of these judgments.

To the conclusion that:

(2) That controlled processing reflects the distinctively *utilitarian character* of these judgments.

In what follows, I shall argue that this controlled processing is unlikely to have much to do with the utilitarian content of these judgments, and that the connection between controlled processing and utilitarian judgment is merely superficial, and thus that even the Modest Model above is at best highly misleading. The Grander claim therefore lacks even the little support it currently seems to have.

### 2.3. Utilitarian Reasoning in Footbridge?

Greene's dual process model assumes that

1. **DP2.1** Utilitarian judgments are generated by controlled processing reflecting utilitarian reasoning/cost-benefit analysis.

But what does Greene mean when he speaks of ‘utilitarian reasoning’ (or ‘cost-benefit analysis’) leading, say, to the minority judgment that it is morally appropriate to push the stranger in Footbridge?

Greene does not, of course, think that individuals making such judgments explicitly endorse Act Utilitarianism or any similar ethical theory. Neither is it plausible that they endorse such a theory implicitly, since virtually all of them make *some* deontological judgments in *some* contexts. Still, if they are to be said to be making ‘utilitarian’ judgments even in the thinnest sense, their *reason* for judging that it's appropriate to push the stranger must be that this would lead to better impartial consequences. And if they reach this conclusion by explicit reasoning, as Greene holds, then they must be reasoning from a corresponding general moral principle. This means that such individuals must be following something at least approximating the following piece of reasoning:^12^

(1) We are required to impartially maximise wellbeing (*The Principle of Utility*).
(2) 5 lives > 1 life.

Therefore,

(3) We are required to sacrifice 1 to save 5.

This would indeed be a piece of reasoning that *sets out* from a utilitarian premise to a utilitarian conclusion—though I'll later question whether it's usefully called utilitarian *reasoning*.

The problem is that this piece of reasoning goes beyond what is plausible to ascribe to non-philosophers who make ‘utilitarian’ judgments in Footbridge or similar cases.

First, although the judgments of such subjects do appear to aim to reduce overall harm (viz. engage in ‘cost-*cost* analysis’), there is no evidence at all for thinking that they have the utilitarian aims of impartially *maximizing* the balance of *benefit* over harm—a very different and far more ambitious aim. Unlike Act Utilitarianism, common-sense morality marks a normative distinction between harm and its prevention, and benefit and its promotion (Ross, 1930/2002). It seems plausible that most subjects accept this distinction. To show that subjects are really aiming to maximize *overall utility*, we would need scenarios where significant harm to one is needed to produce significant positive *benefit* to a greater number—not survival from gruesome death, but increased happiness and flourishing.^13^ To my knowledge, this type of case hasn't yet been studied, but I predict that the rates of ‘utilitarian’ responses to it would drop dramatically even compared to the low rates of such responses to Footbridge. Indeed, it is even doubtful that subjects aim to *minimize* (as opposed to *reduce*) overall harm when they make such utilitarian judgments, for this would imply that they would also judge that we should push the stranger to save even just two innocent lives, a judgment that I predict would be extremely rare at best.

Second, in the large literature looking at Footbridge and similar cases, the small minority that does not judge that we must not sacrifice the one almost always judges that it's merely *permissible*, not *required*, to push, when they are actually allowed to make this distinction (cf. Cushman *et al*., 2006).^14^ Greene's own studies obscure this point because they ask whether it's ‘morally appropriate’ to push, which is ambiguous between permissibility and requirement (Kahane and Shackel, 2010). The problem is that Act Utilitarianism tells us that we *required* to act in the way that maximized well-being. It does not leave any space for a category of supererogatory acts that are morally better, permissible, but *not* required—indeed the view that it is merely permissible for us to maximize impartial good is actually an influential *non-utilitarian* view (Scheffler, 1994).

Once these distinctions are taken into account, we get a somewhat different piece of reasoning:

(1b) We have reason to reduce overall harm (or prima facie *Duty to Save*).
(2) 5 lives > 1 life.

Therefore,

(3b) It is *permissible* to sacrifice one to save 5.

If this is correct, then neither the premises nor the conclusion of this piece of reasoning are really properly utilitarian, *even* in the looser sense of this term used by Greene. That is, these subjects are not really judging in line with utilitarianism even in a *given* context—the judgment they are making is actually incompatible with utilitarianism, and it is based on a moral principle that draws a normative distinction that utilitarianism rejects.

### 2.4. Controlled Processing of What?

This is a genuine problem, but I want to bracket it for the moment because I want to draw attention to an even greater gap in Greene's dual process model. Let's suppose for the moment that subjects are really engaged in the piece of reasoning Greene ascribes to them. Greene's main claim is that when subjects engage in such reasoning and reach utilitarian conclusions, they uniquely do so using controlled processing. This is what the empirical evidence cited above is supposed to show.

But we should ask: what does this controlled processing reflect exactly?

#### 2.4.1. Recognizing a Foundational Principle?

If the controlled processing reflected recognition of the moral principle that we are required to maximize utility (premise 1) then it would perhaps reflect distinctly utilitarian thinking. But this is highly implausible. Even if someone reached this normative conclusion through effortful reflection when first confronted with, say, the Footbridge case, surely this is something they only need to do *once*, so this anyway couldn't account of the greater levels of controlled processing found in responses to a large set of dilemmas. In any case, this is a *foundational* moral principle—it can be a premise for reasoning, but it's not itself plausibly supported by inference. So to the extent that the controlled processing is supposed to reflect *reasoning*, it can't reflect the non-inferential judgment (even: *intuition*) that we should maximize utility.

#### 2.4.2. Counting the Numbers?

Another possibility, which is often implied by the way Greene and others describe utilitarian judgment, is that the controlled processing reflects the calculation of ‘5 lives > 1 life’ (premise 2).

The first problem with this suggestion is that this *isn't* any kind of utilitarian reasoning. This is just standard *non-moral* reasoning, used to apply a moral principle—*any* kind of moral principle.^15^ It is easy to vary the degree of non-moral reasoning needed to apply different moral considerations in a given context. Some ‘utilitarian’ questions involve little or no calculation: ‘Should we give someone in agony a painkiller?’ Others require greater cognitive effort: ‘Should we sacrifice 1 life to save 2x3-4/2+1 lives?’ But then, this is also trivially true of some deontological questions, for example, ‘Should someone break a promise to a friend that conflicts with three promises to his daughter, when this requires lying twice?’

Responses to these three questions are likely to differ in reaction times or DLPFC activation, reflecting these differences in complexity. But of course these processing differences would tell us absolutely nothing about the psychology underlying these moral principles. Non-moral reasoning is simply irrelevant to that question.^16^

So if the controlled processing reflected this calculation, it would merely be a contingent artefact of the particular stimuli Greene and others are comparing, rather than any kind of interesting feature of utilitarian versus deontological thinking. This is why is it misleading to describe *even* the reasoning that Greene (implausibly) ascribes to subjects as ‘utilitarian reasoning’.

But in any case, it is extremely unlikely that the controlled processing reflects this simple calculation. Deontologists are not numerically challenged. It is near certain that *all* subjects considering Footbridge and similar dilemmas make this simple calculation, whether or not they reach a ‘utilitarian’ conclusion. It is astonishing that so many researchers seem to think otherwise.^17^

### 2.5. So What Does the Controlled Processing Reflect?

#### 2.5.1. Overcoming Conflict

If ‘utilitarian’ judgments in Footbridge involve controlled processing then that processing couldn't reflect ‘utilitarian reasoning’. So it must reflect *something else*. What could that be?

Greene's own account suggests a third option. After all, his account says that individuals not only engage in such ‘utilitarian reasoning’, but also need to actively suppress the pre-potent emotional response pushing them in the contrary deontological direction:

1. **DP2.2** When deontological intuitions are present, effortful controlled processing is needed if one is to arrive at a contrary utilitarian judgment.

This is supposed to be why we find greater activation in the dorsal Anterior Cingulate Cortex (dACC), an area associated with conflict, when subjects make utilitarian judgments. As Greene often points out, the dACC is also active in the Stroop paradigm, where controlled processing is needed to overcome the pre-potent impulse to name the colour perceived rather than the written colour word (Greene *et al*., 2004, p. 390).

I have so far deliberately ignored this part of Greene's theory. Greene typically presents the controlled processing associated with utilitarian judgment as reflecting both ‘utilitarian reasoning’ (DP2.1) *and* the conflict generated by the contrary emotion (DP2.2). The argument so far shows that it can *at most* reflect DP2.2.

The problem is that talk about emotional conflict and overcoming an emotional response is ambiguous. DP2.2 can be understood in two ways. The first interpretation, which is implicit in Greene's discussion, doesn't really support his model, and is also implausible. The second, more plausible interpretation is simply incompatible with Greene's overall theory.

I will consider each interpretation in turn.

#### 2.5.2. Inhibiting a Pre-potent Emotion?

The picture implied by Greene's discussion, and simply assumed by others, is given by the analogy to the Stroop paradigm: individuals are making an effort to resist a spurious distorting influence (see Paxton and Greene, 2010). On this view ‘utilitarian’ judgments in Footbridge are the result of something like the following deliberative sequence:

(1) We are required to impartially maximise wellbeing.
(2) 5 lives > 1 life.
(3) Our intuitive aversion to directly killing the one is *spurious*, without *any* moral weight, and therefore should be resisted.

Therefore (with effort),

(4) We are required to sacrifice the one to save 5.

Such rejection of intuitions is of course common in genuine utilitarian thinking for the simple reason that utilitarians reject *all* non-utilitarian moral considerations, and thus all of the intuitions that (at least sometime) drive them. However, there is nothing distinctly utilitarian in rejecting some intuition as spurious—this is something that both utilitarians and deontologists do. Indeed, we also reject intuitions in this way in many non-moral contexts. In any case, since this is a purely negative operation, it tells us nothing *positive* about the source and nature of utilitarian thinking. Instead of the claim about utilitarian judgment in DP2.2, we get the unsurprising claim that whenever strong intuitions are present, effortful controlled processing is needed if one is to arrive at a judgment contrary to these intuitions. This near truism cannot serve the required role in the theoretical and normative constructions to which Greene and others want to put the dual process model to use (Greene, 2008; Singer, 2005).

In any event, this interpretation of the data is also highly implausible. First, if subjects don't even take there to be a legitimate deontological constraint against the intentional killing of an innocent person, why do most of them think it's only permissible, rather than *required*, to kill the 1 to save 5? Indeed, this point suggests that judgments in Footbridge are the product of the *normative interaction* of opposing deontological and ‘utilitarian’ considerations rather than, as on Greene's model, of two independent tracks that are merely in *psychological* conflict with each other. I shall later provide further evidence in support of this claim.

Second, if subjects rejected their aversion to intentionally killing an innocent person as expressing any kind of genuine moral reason, then they should surely reject it as such a reason *quite generally*. But few (if any) subjects make consistently utilitarian decisions across the board even in the restricted context of trolley-style dilemmas. Are subjects simply ‘overcome’ by an emotion they take to be spurious, as people sometimes get confused in the Stroop paradigm? This is utterly implausible.

#### 2.5.3. Deontological Weighing of Duties

The subpersonal talk about conflict generated by a contrary emotion is also compatible with another, and far more plausible, picture of the deliberation that most non-philosophers go through when they make ‘utilitarian’ judgments in Footbridge. They start, as I suggested earlier, with a moral reason or principle:

(1) We have reason to reduce harm (or prima facie *Duty to Save*).

And they count the numbers:

(2) 5 lives > 1 life.

But they also recognize that:

(3) There is reason not to intentionally harm an innocent person (or prima facie *Duty Not to Harm*).

And at least some of them conclude, after some reflection, that:

(4) In *this context*, there is *more* reason to minimize harm.

Which is why they conclude that:

(5) It is permissible to sacrifice one to save 5.

If *this* is what is going on, then not only it is misleading to describe this as ‘utilitarian reasoning’, but what we have here is really a paradigmatic example of a distinctly *deontological* form of deliberation (Ross, 1930/2002). After all Act Utilitarianism leaves *no* space for such *weighing* of competing moral duties or reasons: on Act Utilitarianism (understood as a decision-procedure) there is only a *single* duty, stated by the Principle of Utility, and its application to different contexts.^18^

Greene often portrays deontological responses as simple judgments that some act is absolutely wrong, which he contrasts with the sophisticated utilitarian weighing of competing concerns (Greene, 2008, p. 64). But this gets things exactly upside down. Except at its crudest, deontological thinking involves precisely the weighing of competing moral considerations (including considerations about consequences), whereas utilitarian deliberation leaves space *only* for the (non-moral) comparison of the causal consequences of different lines of action. And, to repeat, in cases like footbridge this utilitarian calculation should be obvious and effortless.

Notice further that Greene and others assume that if controlled processing plays a part in generating these ‘utilitarian’ judgments, then this means that this controlled processing is generating these judgments *from scratch*, and that it does so by *inference*—as if the only alternative is for the controlled processing to produce mere rationalization of some pre-existing emotion or intuition. But controlled processing can play a genuine causal role in producing moral judgments without necessarily being the source of their content.

As described above, controlled processing can help decide between competing *pro tanto* moral reasons (or ‘prima facie duties’^19^ by generating an *all-things-considered* judgment about what ought to be done.^20^ And that decision process can be conscious and effortful without being the result of *inference*—after all, the judgment that, in some given context, one duty *outweighs* another is almost certainly *also* based in intuition! (Ross, 1930/2002.)^21^

This gives us an alternative explanation of the role of controlled processing in utilitarian judgment:

Utilitarian judgments are generated by controlled processing reflecting deliberation about the proper weighing of various moral reasons/principles—some or all of which are deontological in character—when these issue incompatible verdicts about a given situation.^22^

Since research has so far ignored this possibility, it's hard to say with confidence how such deliberation proceeds.^23^ But one plausible possibility is that what we have here is the familiar *deontological* idea that there are *thresholds* of degrees of harm that, when crossed, can defeat deontological constraints. Perhaps torture is absolutely forbidden but most deontologists hold that lying, and even some forms of killing, are permitted when the alternative is extremely bad. Nichols and Mallon (2005) have shown that most people have such thresholds, even though for many of them it is crossed only when a catastrophic outcome is at stake. Individuals who make ‘utilitarian’ judgments in Footbridge might simply be people who have a very low threshold compared to the majority (set at, say, 5 rather than 50 lives), but whose moral thinking is not otherwise especially different.

Notice that this explanation is compatible with DP2.2. In many cases, one of the opposing moral reasons or principles can be especially salient, and dominate, even pre-empt, deliberation. It might thus take some effort to see and seriously consider competing considerations that are less salient. But I will now suggest a further reason why such ‘utilitarian’ judgments are especially difficult.

#### 2.5.4. ‘Utilitarian’ Judgment ≠ Utilitarian Reasoning

Here is another way of making the same point. Think of Bernard Williams's famous anti-utilitarian example of Jim and the Indians, where Jim is told that if he shoots one Indian, the lives of several others would be spared (1973). The point that Williams wanted to make with this example *wasn't* that we *shouldn't* make the ‘utilitarian’ decision to save more lives—he was actually inclined to think we should. Williams's point was rather that this decision shouldn't be as *easy* as is implied by Act Utilitarianism; it should be a *difficult*, *agonising* choice. And indeed, unlike some brain-damaged patients which (as we shall see in the next section) do find this an easy choice, most healthy individuals seem to find such ‘utilitarian’ choices difficult in *exactly* the way Williams described—not merely because we need to overcome some irritating but spurious opposing ‘pre-potent response’.

Greene *et al*. (2004) report that utilitarian judgments were associated with greater activation in the insula, which they speculate might reflect repugnance at the sacrifice of the one. Moretto *et al*. (2009) report that a strong emotional reactions (reflected in skin conductance response) *followed* utilitarian judgments. And Choe and Minn (2011) report that utilitarian judgments were most strongly associated with feeling guilt. These three findings fit Williams's (1966) remarks about the *moral residue* we feel when we are tragically forced to choose one of two profoundly bad options. These findings are hard to square with the common assumption that subjects view the deontological intuition as merely a gut reaction that needs to be resisted.^24^

The lesson here is that it's a mistake to assume that if a moral judgment is ‘utilitarian’ (in the weak sense that, in a given moral context, it favours the act that maximizes utility), then this implies that the deliberation that generated it is also ‘utilitarian’. Quite the opposite: such judgments are typically based in a distinctly deontological form of deliberation that is profoundly *incompatible* with utilitarianism.^25^

I've argued that controlled processing in ‘utilitarian’ judgment in Footbridge reflects, not endorsement of the Principle of Utility, or some simple calculation. Not even the effort to resist a spurious gut reaction. It rather reflects a distinctly deontological form of deliberation to what is only misleadingly called a ‘utilitarian’ conclusion—indeed a conclusion that may itself be based on an intuition about the weighing of competing moral considerations. And this means that, contrary to what has been assumed by current research, this controlled processing tells us *nothing* about the source or nature of utilitarian judgment. If anything, it *only* tells us something about *deontological* deliberation—about the neural processes involved in weighing competing deontological principles.

### 2.6. An Empirical Prediction: Automatic Utilitarian Judgments

If the controlled processing associated with utilitarian judgment in Footbridge-style cases doesn't reflect ‘cost-benefit analysis’, then this suggests that it might be possible to do such an analysis, and arrive at a ‘utilitarian’ conclusion, *without* engaging controlled processing—that is, that it need not require any kind of explicit conscious *reasoning*.

And indeed there is evidence that controlled processing *isn't* always needed to make the judgment that it's morally appropriate to push in Footbridge. As we have seen, a key bit of evidence for the dual process model is that patients with VMPFC damage exhibit greater rates of ‘utilitarian’ judgment. As noted above, this is taken to be evidence for the role of emotion in generating deontological judgment, and so, at most, only evidence for DP1, the first half of Greene's dual process model. But at the same time, it is *also* evidence that ‘utilitarian’ judgments *needn't be* slow and effortful—and thus also potentially evidence against the more important *DP2*. This is because these patients make these judgments very quickly, as a recent study demonstrates. Moretto *et al*. (2009) report *lower* RTs for ‘high conflict’ utilitarian judgments in VMPFC patients compared to healthy controls.^26^

It's also worth noting here that the relevant area of the DLPFC (the area that was reported to be associated with utilitarian judgment in Greene *et al*., 2004) is *damaged* in many of these VMPFC patients (Moll and de Oliviera, 2007). Since other parts of the DLPFC are intact, this doesn't itself show that they are incapable of controlled processing, or that they are not engaged in such processing when making utilitarian judgments. What this *does* strongly suggest is that, if the DLPFC activation reported in Greene *et al*., 2004 reflects the controlled processing healthy subjects engage in when they make utilitarian judgments in Footbridge, then this controlled processing *couldn't* reflect a utilitarian cost-benefit analysis, since these patients can reach this utilitarian conclusion without engaging this brain area. Notice that this proposal is empirically testable: it predicts that cognitive load *won't* affect utilitarian judgment in these patients as it affects healthy ones.

This evidence from VMPFC damage shows that ‘utilitarian’ judgments in Footbridge-style dilemmas *can* be extremely quick and effortless (and, moreover, needn't involve the DLPFC activation reported in Greene *et al*., 2004).^27^ In these cases, it seems doubtful that we should associate these ‘utilitarian’ judgments with effortful deliberation, since they appear to meet standard criteria for automatic processing.

In other words, the standard marks of controlled processing disappear when subjects no longer engage in the (non-utilitarian) deliberative process of weighing competing moral reasons—they disappear *precisely* when subjects more closely approximate a *genuinely utilitarian* mode of thinking!^28^

## 3. New Empirical Evidence: Counterintuitive not Utilitarian

I have offered an alternative interpretation of the existing evidence: the controlled processing associated with so-called utilitarian judgments in Footbridge style cases really reflects deliberation that has nothing to do with utilitarianism proper, and is indeed incompatible with it. I now want to briefly review new evidence from a recent neuroimaging study we have done that directly supports my alternative interpretation, and also offers a significant further challenge to Greene's dual processing model.

### 3.1. The Problematic Inference: An Overlooked Confound?

Consider again the problematic inference I've mentioned above from evidence associating ‘utilitarian’ judgments with controlled processing, to the conclusion that this processing reflects the distinctive utilitarian character of these judgments. Here is another way of bringing out this point. Greene and other researchers often compare utilitarian judgments that are strongly counterintuitive (such as to push the fat man) with contrary deontological judgments that are strongly intuitive (e.g. don't push). They observe various behavioural and neural differences between the two. And they conclude that these differences reflect differences between utilitarian and deontological judgments. But there is a glaring alternative explanation they have overlooked: these differences might simply reflect differences between *counterintuitive* and *intuitive* judgments, quite *regardless* of their content. If this is correct, then, again, the apparent tie between process and content is really just an artefact of the kinds of scenarios that researchers have studied, reflecting nothing deep about utilitarian and deontological judgments.

**Figure 1:**
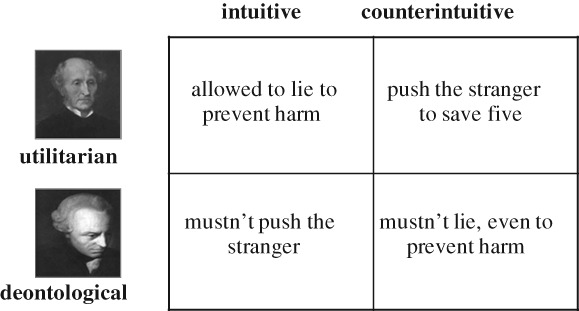
Intuitiveness and content as distinct variables in moral judgment.

Now this wouldn't be the case if all counterintuitive judgments were utilitarian, and all intuitive ones deontological. But this is plainly not so, as Kant's notorious assertion that we mustn't lie even to prevent murder demonstrates (Kant, 1797/1966; this example is mentioned in Greene, 2008). Most people seem to find it immediately *obvious* that we should lie in such a case—it is natural to say that they find this ‘utilitarian’ decision *intuitive*, and the contrary deontological one strongly *counterintuitive*. And the mere existence of these overlooked pairings of content (utilitarian/deontological) and intuitiveness (intuitive/counterintuitive) already presents clear counterexamples to what I called Greene's Grand Model.

Could Greene deny that there really are these counterexamples? With respect to supposed instances of intuitive utilitarian judgments, he might argue that although some ‘utilitarian’ judgments are immediate and effortless, and based on intuition in some broad sense, they are nevertheless not genuinely automatic. This move has little plausibility. In any case, Greene himself speculates that utilitarian judgments have their origin in immediate affective responses to harm to others (Cushman, Young and Greene, 2010). So he already seems committed to accepting this counterexample, although it's not clear how exactly to square this with his general model.

I'm more interested, however, in the possibility of counterintuitive deontological judgments, since they challenge the crucial tie between controlled processing and utilitarianism. How might Greene challenge this possibility? Greene doesn't deny that some deontological claims, such Kant's remarks on lying, are counterintuitive. But he seems to treat these as rare cases where a commitment to an explicit philosophical theory leads some philosophers to make counterintuitive judgments (Greene, 2008, pp. 65-66; Paxton and Greene, 2010). However, non-philosophers plainly make similar deontological judgments, and Greene is surely committed to claiming that such judgments must involve utterly different psychological processes than the counterintuitive utilitarian judgments on which the research has so far focused. One possibility that is in line with Greene's model is that judgments that appear to be counterintuitive deontological judgments really reflect, not the effortful overcoming of a utilitarian intuition, but unusual affective responses—e.g. an atypically strong aversion to lying.

In any case, apparent everyday examples of counterintuitive deontological judgments, and utilitarian judgments based on intuition, have so far been strangely ignored by researchers. What would we find if we examined them?

Greene's dual process model makes clear predictions. Utilitarian judgments should involve controlled processing, whether or not they are (in the loose sense set out above) intuitive or not. And the neural and behavioural correlates of counterintuitive deontological judgments should be utterly different from those of counterintuitive utilitarian ones such as in Footbridge. (If Greene's model doesn't make these predictions, what predictions *does* it make?) The alternative hypothesis I've just offered makes exactly contrary predictions: counterintuitive judgments should engage similar brain areas whether or not they are deontological or utilitarians; and a parallel prediction follows for intuitive judgments. In a recent neuroimaging study, we set out to test these competing hypotheses (for full details, see Kahane *et al*., 2012).

### 3.2. Intuitiveness versus Content

We operationalized ‘intuitiveness’ by surveying the unreflective judgments of an independent sample of non-philosophers, and classifying types of judgments as intuitive if they were clearly dominant—if they were made unreflectively by 13 or more out of the 18 independent judges; the contrary judgments were classified as ‘counterintuitive’.^29^ In addition to the commonly used dilemmas such as Footbridge where the utilitarian choice is typically counterintuitive, we also used dilemmas, not previously studied, where the deontological choice is counterintuitive, such as refusing to lie to prevent harm. Importantly, although only a minority of these lay judges endorsed refusal to lie in such cases, it is highly unlikely, to put it mildly, that this refusal was based in adherence to some explicit deontological theory.

A separate group of subjects was then placed in an fMRI while they responded to dilemmas of both kinds. Our main finding was that the apparent neural and behavioural differences between utilitarian and deontological judgments in trolley-like dilemmas are driven almost exclusively by differences in their intuitiveness, not in their content (see Kahane *et al*., 2012). To focus on the case that is most relevant to our discussion, ‘utilitarian’ judgments such as that it is appropriate to push in Footbridge, and what we can call ‘ultra-deontological’ judgments, such as refusing to lie to prevent harm, were associated with strikingly similar patterns of neural activation, compared to contrary intuitive judgments. In other words, judgments with *radically opposing* contents were based in similar neural processes.^30^

It's worth noting we didn't find that counterintuitive judgments were associated with greater response times, or with activation in classical cognitive areas such as the DLPFC and the parietal lobe, compared to contrary intuitive ones. And, importantly, we did not find this even when we compared only counterintuitive utilitarian judgments with intuitive deontological ones—the comparison closest to that in Greene *et al*., 2004.^31^ We did, however, find that counterintuitive judgments were associated with greater perceived difficulty, and with activation in the subgenual part of the rostral ACC, an area that has been implicated in affective conflict (Etkin *et al*., 2006)—but also in feeling guilt (Zahn *et al*., 2009a; Zahn *et al*., 2009b; Green *et al*., 2010).^32^ So we found only rather qualified support for the hypothesis that counterintuitive judgments are generally associated with controlled processing. These neural activations seems most likely to reflect—as I suggested above—the process of deliberating about competing moral considerations rather than explicit moral reasoning to a new conclusion.^33^

These results are incompatible with Greene's dual process model. They show, first, that DP1 and DP2 are not generally true, as the ‘grand’ version of the model asserts. But our results also show that the differences between utilitarian and deontological judgments largely reflect differences in intuitiveness even when we consider *only* Footbridge-style cases. So these results cast doubt even on the modest version of these claims. So, again, it seems that the neural and behavioural correlates of paradigmatic ‘utilitarian’ judgments merely reflect deliberation to a counter-intuitive conclusion—and not anything distinctively utilitarian.

What we get, instead of Greene's exciting and controversial dual-process model, is an alternative and rather less surprising dual-process model:

Intuitive judgments are generally associated with automatic processing, and counterintuitive judgments with controlled processing.

Let me quickly qualify this proposal in some obvious ways. This is a claim about the broad processes (‘automatic’ and ‘controlled’) associated with intuitive versus counterintuitive judgments. But it's of course almost certain that at a finer grained level we will find differences in the processes that underlie judgments with different contents—not just ‘utilitarian’ versus deontological, but also different sub-types of such judgments. In particular, ‘automaticity’ is a broad-brush category. It's at least plausible that different kinds of intuitions would be triggered by different factors, and might themselves involve rather different processes.^34^

In personal communication, Greene suggests that his dual process theory is really meant to essentially make this more general claim about intuitive and counterintuitive judgment. This, however, is certainly not how the theory has so far been presented or understood. If Greene's claim is simply that many utilitarian judgments are counterintuitive, and that the processes involved in making such judgment merely reflect the processes generally involved in making counterintuitive judgments, then, again, these are near truisms that cannot support the ambitious theoretical and normative arguments that Greene and others have defended on the basis of the theory (they imply nothing interesting about utilitarianism, let alone anything *favourable* to utilitarianism). In addition, the independent argument of the previous sections makes the stronger claim that these controlled processes in fact involve a form of deliberation that is incompatible with utilitarianism, and thus simply *incompatible* with these further arguments.^35^

It might be replied that even if controlled processing is generally associated with counterintuitive moral judgments, such processing nevertheless *favours* utilitarian judgments. If this is a general claim, not specific to trolley-like cases, it's hard to see what evidence is supposed to support it. How exactly are we supposed to measure such a general tendency? In any case, if the claim is simply that utilitarianism issues more counterintuitive conclusions compared to many other moral theories, then again this is a mere truism. And there is again the point that many of these ‘utilitarian’ judgments are really due to non-utilitarian deliberation. Finally, in the next section I will suggest that we don't yet have sufficient evidence for thinking that controlled processing favours utilitarian judgment *even* in trolley-like cases.

### 3.3. Controlled Processing and Counterintuitive Judgment

It isn't surprising that counterintuitive judgments are more likely to involve a measure of controlled processing. I want to end by highlighting another faulty inference: the mistake of thinking that evidence showing that counterintuitive judgments typically involve controlled processing implies that controlled processing typically leads to counterintuitive conclusions. But this second claim doesn't automatically follow from the first.

**Figure 2:**
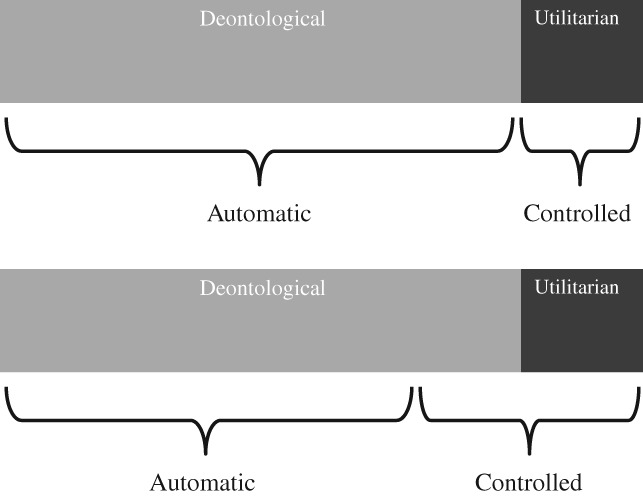
An association between utilitarian judgment and controlled processing need not imply that controlled processing favours utilitarian outcomes.

Consider for example the case of utilitarian judgments in Footbridge-like dilemmas. We know that only a minority of subjects make counterintuitive utilitarian judgments in such dilemmas. And let us suppose that most of these judgments are based in controlled processing, whereas the majority of intuitive deontological judgments are based in automatic processing. Such a result is indeed *compatible* with a strong tie between controlled processing and counterintuitive utilitarian judgment. But it's *also* compatible with the possibility that a clear *majority* of those who engage controlled processing nevertheless end up ultimately endorsing their initial intuition. Evidence for the involvement of controlled processing in such judgments might simply be swamped by the automatic processing that, on the accepted picture, is supposed to drive the majority of deontological judgments (see Figure 2). This is why it's premature to infer, simply from the fact that counterintuitive judgments are largely based in controlled processing, that controlled processing strongly *favours* counterintuitive outputs.^36^

## 4. Conclusion

It is natural to expect that the dual process model that has proven fruitful in many psychological domains will also shed light on moral psychology. What is most striking about Greene's version of the dual process model is that it has tried to tie automatic and controlled processing to distinct types of moral content. In this article, I have tried to clarify Greene's model and the evidence that is taken to support it. I have argued that the controlled processing that has been associated with so-called utilitarian judgments in trolley-like dilemmas *doesn't* reflect ‘utilitarian reasoning’, and tells us little about the source or nature of utilitarianism. It has appeared to do so only because researchers have not been clear enough about what they mean by ‘utilitarian judgment’ and ‘utilitarian reasoning’. Greater conceptual precision, coupled with a closer reading of the existing body of evidence, should lead us to doubt Greene's model. In fact, the instances of controlled processing that have been taken to most strongly support this claim actually turns out to ironically reflect common forms of deliberation that are simply incompatible with a utilitarian outlook.

## Footnotes

For simplicity, I will follow Greene and others in using ‘utilitarian’ to refer to what are more accurately called ‘utility maximising’ judgments (see Kahane and Shackel, 2010).

Paxton and Greene (2010) also discuss cases where individuals may genuinely apply explicit deontological principles using controlled processing, though on Greene's view such principles are themselves the products of past rationalizations of intuition.

In fact, although Greene and other researchers often present the trolley case as a core part of the traditional case *against* utilitarianism, this is highly misleading at best. The trolley problem is problem *internal* to non-utilitarian ethics, as a look at Foot, 1978 and Thomson, 1985 would confirm.

It's common for researchers to report that some study has shown utilitarian judgments to be this or that, where it at most shows this about utilitarian (viz. utility maximising) judgments *in trolley-like dilemmas*. This slide from a narrow modest claim to an unwarranted general claim is unfortunately frequent.

Or more precisely, the factors to which *deontological* intuitions are sensitive; it's obvious enough that utilitarian judgments are sensitive to consequences.

I am glossing here over various problems in identifying intuition, in the philosophers' sense, with immediate automatic processing.

Schwitzgebel and Cushman, 2012 may appear to provide such evidence, but it seems to me to only show that, unsurprisingly, the intuitions of experts can be subject to morally irrelevant influences. But it's perfectly rational (rather than any kind of ‘rationalization’) for these experts to then endorse principles consistent with these intuitions.

It might be objected that the claim that deontological judgment is based in emotion *is* surprising and controversial. It would be to some extent surprising and controversial if it turned out that deontological judgments were generally based on emotion, though something like this is already asserted by influential non-utilitarian authors (see e.g. Williams, 1973). In any case, for the reasons given above, not much revolves on whether we take DP1 to be also a claim about emotion.

But the anatomical labelling of this activation as in the DLPFC is controversial (see Moll and de Oliveira-Souza, 2007). Worse, Tassy *et al*., (2012) found that disruption of the DLPFC using transcranial magnetic stimulation actually led to an *increase* in utilitarian judgment!

However, the association between working memory capacity and utilitarian judgment held *only* for cases where the ‘utilitarian’ option involved harming an individual who would be harmed anyway (Moore *et al*., 2008), a type of choice that even most hard-core deontologists would endorse. The same was true for the inducement of utilitarian judgment using the ‘Cognitive Reflection Test’ (CRT) in Paxton *et al*., 2012, where rates of utilitarian judgment for the Footbridge case were *not* affected (Paxton *et al*. also report a correlation between individual difference in performance on the CRT and utilitarian judgment but only in a variant of Footbridge where failure to push the one would lead to *thousands* of deaths. I discuss cases of such catastrophic harm below.) For all we know at this stage, most current evidence for controlled processing in utilitarian judgment may really be due to this type of dilemma which is not really at issue between utilitarians and their opponents. In addition, the CRT may introduce a number of confounds, since it involves mathematical problems that may bias subjects towards utilitarian solutions, and since it was originally designed precisely to measure individual differences in endorsement of counterintuitive *cost-benefit* solutions.

Greene also cites the example of Haidt's work on the role of disgust in generating intuitions against harmless violations, although these would be examples of deontological intuitions only in the loosest sense. But Haidt reports that the judgments of liberals who rejected these intuitions—and were thus making utilitarian judgments in the relevant sense—were *not* influenced by manipulation of cognitive load (reported in Haidt, 2012).

This picture is essentially endorsed in Cushman, Young and Greene (2010), where they refer to the Principle of Utility as the ‘welfare principle’.

One famous example is William James's (1891) rejection of an entire universe of supremely happy beings if this required even a single child to suffer horrible torture.

This point is made in Kamm, 2010, pp. 339-340. Selim Berker drew my attention to this.

This point is actually supported by Greene's own work: Shenhav and Greene (2010) report that so-called ‘utilitarian’ reasoning has the *same* neural correlates as *non-moral* reasoning.

When Greene (2008) claims that utilitarian judgment must be based in controlled processing because of its aggregative character, he is overlooking this. Notice further that this point isn't already acknowledged by Greene *et al*. (2004) when they describe utilitarian judgments as generated by ‘domain general’ cognitive processes. My point above isn't about the kind of subpersonal mechanisms that underlie utilitarian judgments; for all we know, distinctly moral forms of reasoning might be subserved by domain general processes. In any case, if the controlled processing really involves no more than the application of some general principle (of whatever content), then DP2 would be either false or highly misleading.

I wrote that deontologists aren't numerically challenged—but in fact virtually all subjects make a mixture of ‘utilitarian’ and deontological judgments. Are we to assume that they count the numbers only when they make utilitarian judgments? There is the further worry that it's doubtful that judging that 5 is greater than 1 really requires *any* kind of cognitive effort.

In addition, an unpublished study by Shenhav and Greene suggests that the VMPFC is involved, not in generating affective responses driving deontological judgment, but in *integrating* such affective responses (correlated with amygdala activity) and calculations of utility (Greene, personal communication). This integration clearly reflects the kind of weighing of duties I've described. It would make no sense if subjects saw the contribution of the deontological-affective component as entirely spurious, because on this picture our moral judgments should be completely insulated from the spurious emotional response, whose strength should make no difference at all.

I follow W. D. Ross in speaking of ‘prima facie duties’, but the relevant reasons are not literally ‘prima facie’ (merely appearing to be genuine reasons), and are best described as ‘*pro tanto*’ (genuine reasons that are outweighed in a context).

Nichols and Mallon (2005) show that non-philosophers can easily distinguish between whether an act violated some rule (weak impermissibility) and whether that act was wrong, *all things considered*.

It might be objected that subjects can't be engaged in such deliberation because, although something like the principle of utility is an explicit principle that people can apply, the resistance to, e.g., pushing the stranger in Footbridge is an intuition that most people are unable to articulate as an explicit principle (Cushman *et al*., 2006). But this is irrelevant. We can be conscious of, and torn between, opposing moral considerations even if we can't articulate them as fully explicit principles.

Notice that I'm not claiming that *no one* treats the intuition not to push as spurious, only that most people don't. There certainly *are* clear cases where people reject their intuitions/affective reactions are simply morally spurious—one example is the way liberals view their disgust reactions to harmless violations (Haidt, 2001).

Stanovich (2009) argues that talk about controlled processes fails to distinguish between *algorithmic* processes (such as explicit calculation or inference) and what he calls *reflective* processes, which relates to control states that regulate behaviour at a higher-level. The kind of deliberation described above would fall on the reflective side of this division.

These findings suggest that many subjects not only accept as valid both of the competing moral considerations in trolley dilemmas, as I have argued earlier, but that they might even hold (implicitly if not explicitly) that these dilemmas are what philosophers call ‘*true dilemmas*’—that *both* options in these dilemmas are wrong. This fits both the point mentioned earlier, that people tend to see the utilitarian choice as permissible rather than required, and the recent study reporting that subjects rate both options as somewhat wrong, when allowed to do so (see Kurzban *et al*., 2012; subjects did rate pushing in Footbridge as *more* wrong).

This is why it's misguided to suggest, as Greene does, that we take ‘utilitarian’ to refer to whatever processes turn out to underlie judgments with ‘characteristically utilitarian content’ (Greene, 2008).

Utilitarian judgments in personal dilemmas in healthy subjects were associated with longer RTs compared to deontological ones, but this difference was absent in VMPFC patients—again showing that the increase in response time reflected not ‘utilitarian reasoning’ but the conflict between its conclusion and a contrary deontological pull.

In addition, Greene *et al*. (2008) report that healthy subjects who had a strong tendency to make ‘utilitarian’ judgments in Footbridge (and other high conflict dilemmas) do so *faster* than it takes most subjects to make *deontological* judgments. However, they also report that cognitive load increased RTs in utilitarian judgment even in these subjects.

Besides VMPFC patients, the other group that seems to approach moral dilemmas in a genuinely utilitarian fashion are individuals high on psychopathy (Bartels and Pizarro, 2011).

On this operationalization, ‘intuitiveness’ is a *population-relative* notion, so judging that we are forbidden to push in Footbridge counts as intuitive in this sense even though some individuals (such as VMPFC damaged patients) may lack this intuition. Moreover, recording the unreflective judgments of individuals is only an indirect measure of automaticity, so the classification used left it open what processes underlie judgments classified as ‘intuitive’. More importantly, it left it entirely open what processes underlie judgments classified as ‘*counterintuitive*’.

Moreover, the study did not find common correlates for so-called ‘utilitarian’ judgments across different domains. This further supports the hypothesis that the processes previously ascribed to utilitarian judgment are in fact generic deliberative processes. Judgments we classified as intuitive utilitarian judgments typically involved concern for others' wellbeing that did not involve counting numbers. But as noted earlier, the correlates of such calculations are not of great interest for moral psychology. We did find that deontological judgments of different kinds were commonly associated with activation in the TPJ and PCC. These are not quite the areas Greene has previously associated with deontological judgment. The TPJ is commonly associated with theory of mind, and implicated in ascription of intention in moral cognition, so this activation might just reflect the role of intention in deontological judgment.

Although absence of evidence isn't necessarily evidence of absence, I should note several independent worries about Greene's earlier result. First, Greene *et al*., 2004 relied on the rather peculiar subject-by-subject construct of ‘difficult’ dilemmas, which, they report, typically excluded responses to Footbridge from the analysis—the very dilemma which this study was supposed to target! Second, so far as I know, Greene's DLPFC finding hasn't yet been replicated. Indeed, Shenhav and Greene, 2010, which used a better-controlled set of dilemmas, *also* failed to find an association between utilitarian judgments and DLPFC activation.

Interestingly, patients with damage to the VMPFC also typically suffer damage in this area.

This is also supported by the finding that cognitive load only affects the RTs of utilitarian judgments but *not* their frequency (Greene *et al*., 2008).

Schaich Borg *et al*., (2006) already reported (unsurprising) neural differences between responses to moral dilemmas involving different kinds of deontological considerations. My alternative model is also compatible with some kinds of intuitive judgments being more ‘cognitive’ than others.

Needless to say, if utilitarianism is understood only as a criterion of rightness (and not also as a decision procedure), then it is logically compatible with any form of deliberation—including applying the Categorical Imperative or Rational Egoism. But Greene's claims about the origins of utilitarian thinking *require* treating it, at least in this context, as referring to a distinctive decision procedure.

Of course, even if all experimental subjects who engaged in effortful deliberation about Footbridge ended up endorsing the utilitarian conclusion, this still wouldn't show that this is what controlled processing *favours*. Subjects in experiments have seconds, at most minutes, to reflect on the dilemmas they are given. The initial outcome of reflection is often overturned by further deliberation.
